# DHHC21 deficiency attenuates renal dysfunction during septic injury

**DOI:** 10.1038/s41598-021-89983-x

**Published:** 2021-05-27

**Authors:** Xiaoyuan Yang, Ethan Zheng, Yonggang Ma, Victor Chatterjee, Nuria Villalba, Jerome W. Breslin, Ruisheng Liu, Mack H. Wu, Sarah Y. Yuan

**Affiliations:** 1 Department of Molecular Pharmacology and Physiology, University of South Florida Morsani College of Medicine, Tampa, Florida 33612 USA; 2 Department of Surgery, University of South Florida Morsani College of Medicine, Tampa, Florida 33612 USA

**Keywords:** Infectious diseases, Circulation, Kidney, Post-translational modifications

## Abstract

Renal dysfunction is one of the most common complications of septic injury. One critical contributor to septic injury-induced renal dysfunction is renal vascular dysfunction. Protein palmitoylation serves as a novel regulator of vascular function. Here, we examined whether palmitoyl acyltransferase (PAT)-DHHC21 contributes to septic injury-induced renal dysfunction through regulating renal hemodynamics. Multispectral optoacoustic imaging showed that cecal ligation and puncture (CLP)-induced septic injury caused impaired renal excretion, which was improved in DHHC21 functional deficient (*Zdhhc21*^*dep/dep*^) mice. DHHC21 deficiency attenuated CLP-induced renal pathology, characterized by tissue structural damage and circulating injury markers. Importantly, DHHC21 loss-of-function led to better-preserved renal perfusion and oxygen saturation after CLP. The CLP-caused reduction in renal blood flow was also ameliorated in *Zdhhc21*^*dep/dep*^ mice. Next, CLP promoted the palmitoylation of vascular α1-adrenergic receptor (α1AR) and the activation of its downstream effector ERK, which were blunted in *Zdhhc21*^*dep/dep*^ mice. Vasoreactivity analysis revealed that renal arteries from *Zdhhc21*^*dep/dep*^ mice displayed reduced constriction response to α1AR agonist phenylephrine compared to those from wild-type mice. Consistently, inhibiting PATs with 2-bromopalmitate caused a blunted vasoconstriction response to phenylephrine in small arteries isolated from human kidneys. Therefore, DHHC21 contributes to impaired renal perfusion and function during septic injury via promoting α1AR palmitoylation-associated vasoconstriction.

## Introduction

Septic injury often progresses into multiple organ dysfunction, serving as a leading cause of mortality in the intensive care units^1,2^. Kidneys are one of the most susceptible organs to septic injury. Patients with septic injury-induced renal dysfunction tend to have prolonged hospital-stays, worse outcomes, and significantly higher mortality^1^. The pathogenesis of septic injury-associated renal dysfunction is a complex and multifactorial process; one prevailing mechanism is renal vascular dysfunction^3–5^. Studies of patients and large animals with septic injury-induced renal dysfunction have shown significantly increased renal vascular resistance^6,7^, contributing to decreased renal blood flow (RBF) in septic injury^7–9^. This reduction of RBF has been confirmed in sepsis patients with kidney injury using different techniques including renal Doppler, thermodilution and phase contrast magnetic resonance imaging^10,11^. Regional ischemia and hypoxia caused by insufficient renal tissue perfusion can result in impaired glomerular filtration coupled with tubular epithelial cell death, leading to renal dysfunction^12–14^.

Mechanisms responsible for renal tissue hypoperfusion in septic injury have not yet been fully elucidated. Protein palmitoylation, the covalent attachment of a 16-carbon palmitic acid to the cysteine residue(s) of a protein, has recently been recognized as a novel regulator of vascular function^15,16^. The attached palmitate increases the hydrophobicity of the modified proteins in blood vessels and affects their localization, stability, and conformation, thereby regulating many aspects of vascular function at posttranslational level^17–19^. This reversible modification is catalyzed by a family of palmitoyl acyltransferases (PATs) containing the Asp-His-His-Cys (DHHC) motif^20,21^. So far, 23 PAT-DHHCs have been identified in humans^21^. Recent studies have reported the involvement of PAT-DHHCs in hemodynamic regulation, peripheral arterial disease, and inflammation-induced vascular injury^15,16,22^; however, there is lack of information regarding the roles of PATs in regulating renal vascular function in septic injury.

PAT-DHHC21, unlike the majority of other DHHCs, is expressed on the plasma membrane, making it possible for DHHC21 to regulate the activities of cell surface receptors in blood vessels^18,23^. Our previous findings indicate that DHHC21 functional deficiency demonstrates survival benefits in animals with septic injury^22^. Moreover, a recent study shows that DHHC21 regulates vascular tone through the palmitoylation of vascular α1-adrenergic receptor (α1AR), a seven-transmembrane G protein-coupled receptor (GPCR) that induces smooth muscle contraction^15^. Increased release of endogenous α1AR agonist norepinephrine has been reported in patients with septic injury, contributing to intrarenal vasoconstriction and tissue hypoperfusion^24^. Norepinephrine is also used as the first-line vasopressor to treat septic shock, which could further augment regional ischemia in kidneys^12,25^. Therefore, in this study, we utilized DHHC21-deficient (*Zdhhc21*^*dep/dep*^) mice and tested the hypothesis that DHHC21 contributes to renal dysfunction and tissue damage in septic injury via promoting α1AR palmitoylation-dependent renal vasoconstriction and tissue hypoperfusion. Our findings indicated that *Zdhhc21*^*dep/dep*^ mice were more resistant to septic injury-induced renal dysfunction and structural damage. DHHC21 loss-of-function improved renal perfusion and RBF through inhibiting palmitoylation of α1AR and its ability to mediate renal vasoconstriction. Thus, we suggest DHHC21 as a novel mediator of septic injury-induced renal dysfunction via mechanisms involving α1AR palmitoylation and α1AR activation-associated renal tissue hypoperfusion.

## Results

### DHHC21 loss-of-function reduces renal dysfunction in septic injury

**Figure 1:**
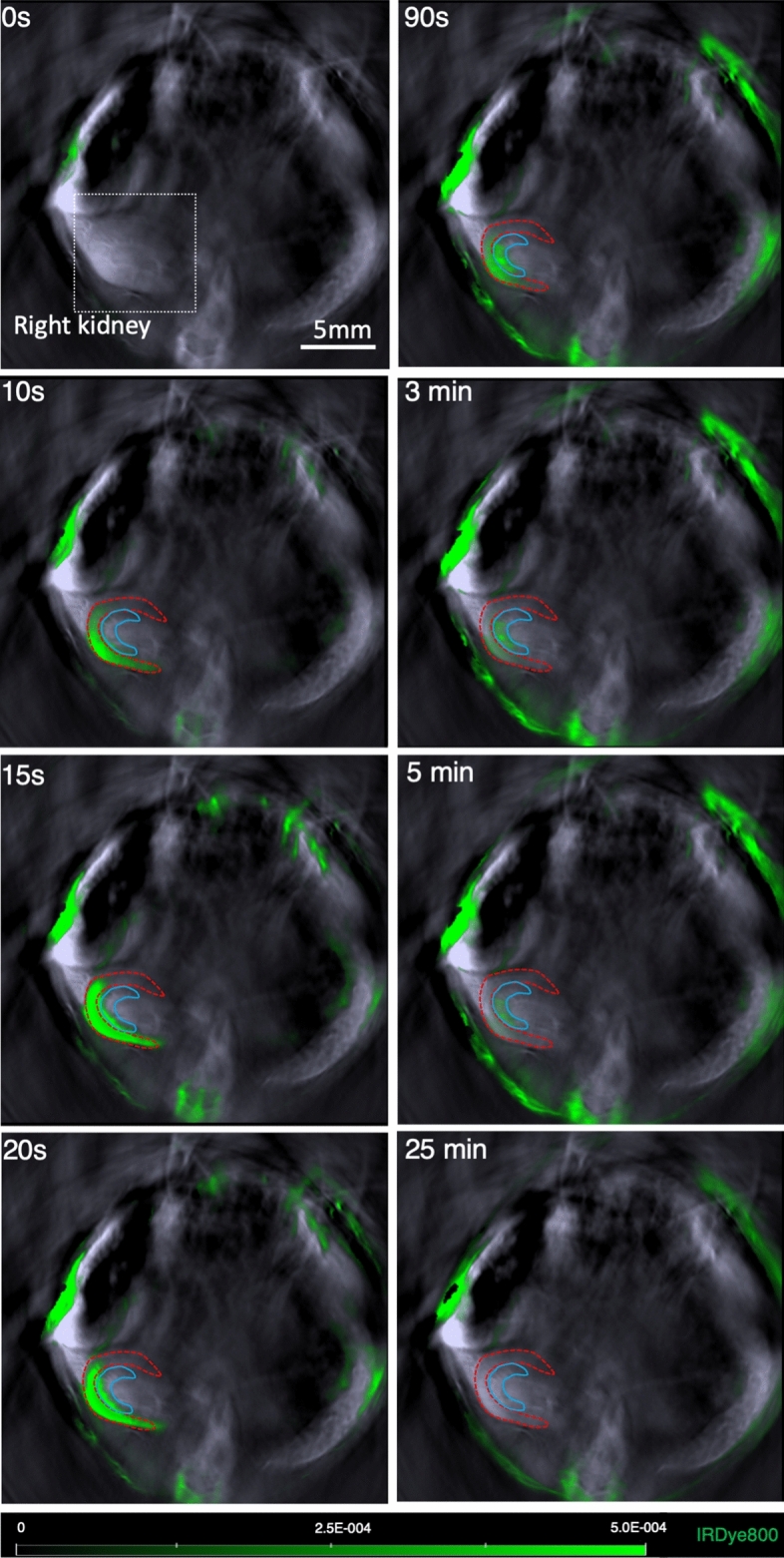
Representative MSOT images showing the clearance of IRDye800CW from mouse kidneys. IRDye800CW (60 nM dissolved in 0.9% saline) was injected intravenously into WT mice 24 h after sham operation. The clearance/movement of IRDye800CW (green) in the cross section of the right kidney (white square) was recorded. ROIs in cortex and medulla/pelvis region of the kidney are delineated in red and blue, respectively. Representative images of 28 mice (n = 7 in each group). The green bar represents the scale for the mean pixel intensity of the IRDye800CW signal.

**Figure 2:**
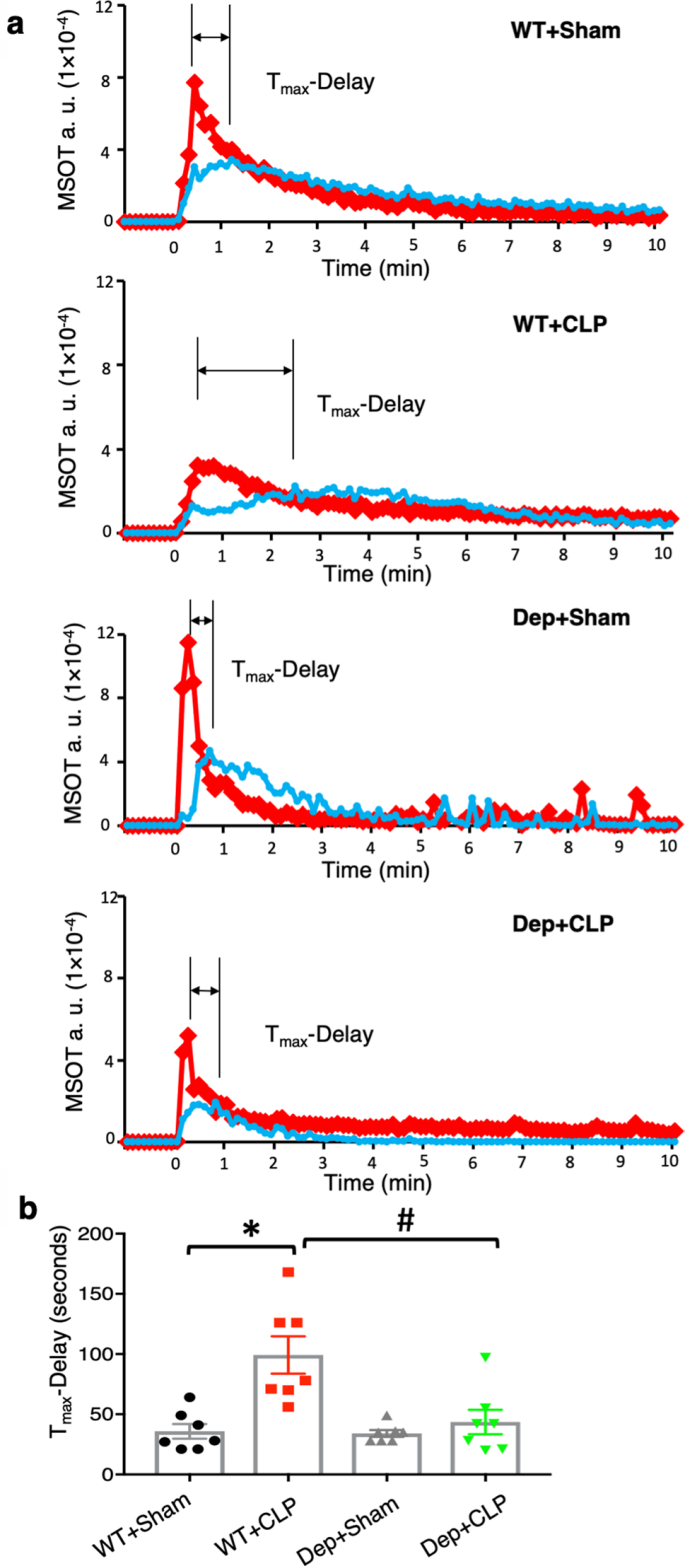
Septic injury-induced delay in renal clearance of IRDye800CW is significantly attenuated in *Zdhhc21*^*dep/dep*^ mice. (**a**) Representative IRDye800CW clearance kinetics of the cortex (red) and medulla/pelvis region (blue). Tmax-delay is the interval between the peaks of pixel intensity in cortex and medulla/pelvis region. (**b**) DHHC21 functional deficiency attenuates septic injury-induced increase in Tmax-delay. Results represent mean ± SEM. n = 7, *p ≤ 0.05 versus WT + Sham, ^#^p ≤ 0.05 versus WT + CLP.

*Zdhhc21*^*dep/dep*^ mice were employed to investigate the role of PAT-DHHC21 in renal dysfunction during septic injury. DHHC21 functional deficiency in *Zdhhc21*^*dep/dep*^ mice is due to a 3 bp deletion in exon 7 of *Zdhhc21* gene, resulting in the loss of a single amino acid phenylalanine (F) at position 233^26^. Detailed information about the genotype and phenotype of *Zdhhc21*^*dep/dep*^ mice is presented in Supplementary Figure S1. We compared renal excretion between WT and *Zdhhc21*^*dep/dep*^ mice using a novel and minimally invasive imaging technique, multispectral optoacoustic tomography (MSOT). MSOT relies on the photoacoustic effect and detects the ultrasound signals produced by the thermal expansion of tissues/molecules when excited by near-infrared (NIR, 680–950 nm) laser pulses^27^. NIR dye IRDye800CW, which is exclusively excreted by the kidneys^28,29^, was injected intravenously 24 h after sham procedure and CLP-induced septic injury. As shown in Fig. 1, real-time recording of IRDye800CW clearance showed that IRDye800CW first appeared at the renal cortex region, then moved towards the medulla-pelvis region before being rapidly excreted by the kidneys. The time interval between the peak intensity of the cortex and medulla-pelvis region (Tmax-delay) was determined based on the clearance kinetics of IRDye800CW as shown in Fig. 2a. Greater Tmax-delay indicates that IRDye800CW takes longer to transit from cortex to medulla-pelvis region, suggesting impaired renal excretion^30^. DHHC21 loss-of-function showed no significant effect on renal excretion in sham controls; however, septic injury-induced increase in Tmax-delay was greatly inhibited in *Zdhhc21*^*dep/dep*^ mice (Fig. 2b).

### DHHC21 functional deficiency attenuates septic injury-induced renal damage

**Figure 3:**
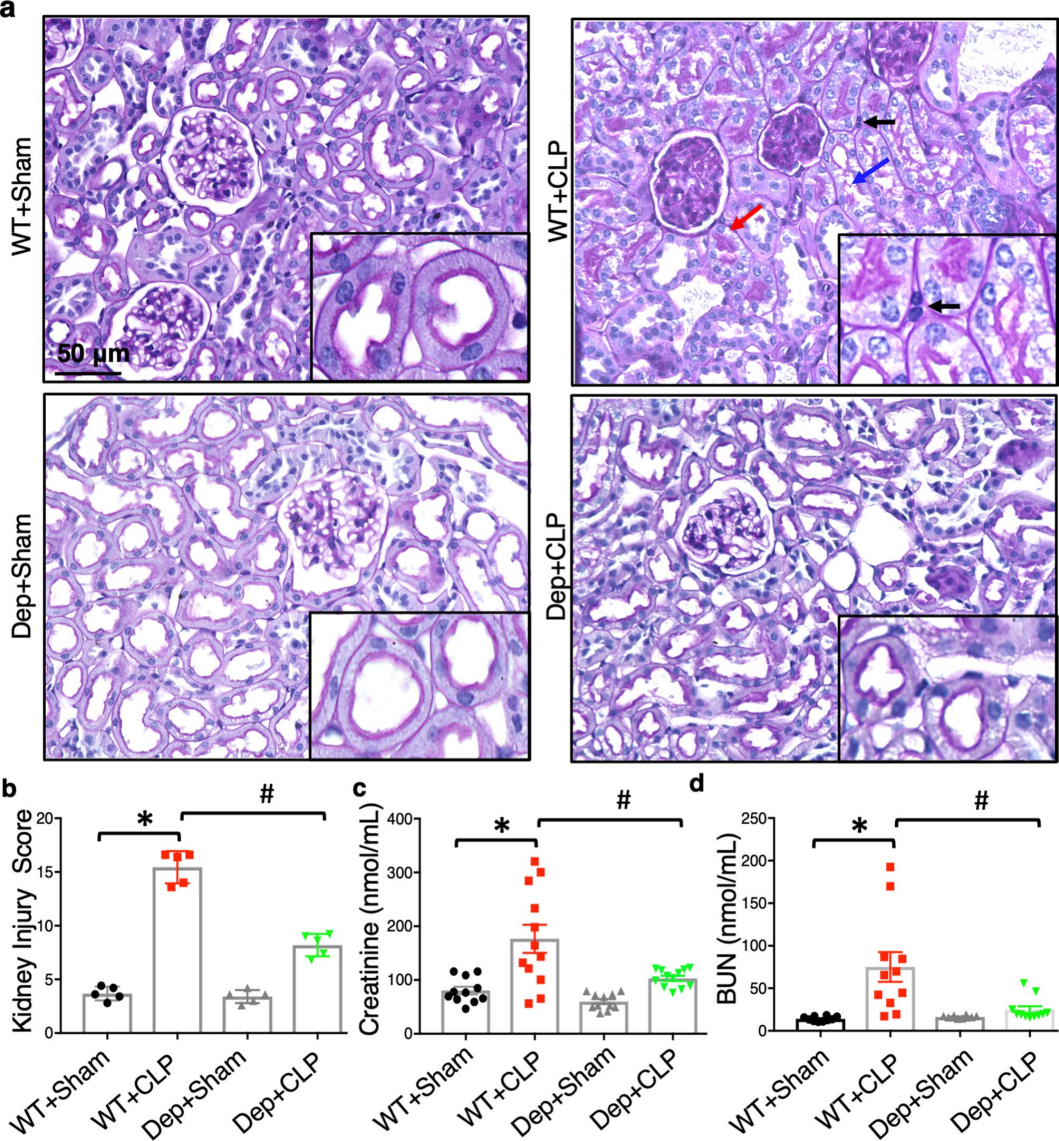
*Zdhhc21*^*dep/dep*^ mice are resistant to septic injury-induced kidney damage. (**a**) Representative images of Periodic Acid-Schiff-stained renal tissue collected 24 h after septic injury induction. Pathological alterations are indicated by arrows with different colors: brush border loss (blue), tubular cell detachment (red), neutrophil infiltration (black). Images are representative of 5 mice. (**b**) Mice with DHHC21 loss-of-function display a reduced renal injury score than WT mice after septic injury. n = 5 independent experiments; 5 views per animal were imaged and analyzed. (**c**) The level of circulating creatinine. Mouse plasma was collected 24 h after CLP-induced septic injury. n = 12. (**d**) The level of blood urea nitrogen 24 h after septic injury. n = 11. Results represent mean ± SEM. *p ≤ 0.05 versus WT + Sham, ^#^p ≤ 0.05 versus WT + CLP.

We then performed Periodic Acid-Schiff staining to visualize kidney structural damage in WT and *Zdhhc21*^*dep/dep*^ mice after CLP. As shown in Fig. 3a, septic injury led to severe renal tissue damage, including glomerular abnormality, loss of brush border in proximal tubule, vacuolation of tubule epithelium, tubular cell detachment/necrosis, and neutrophil infiltration; however, these morphological alterations were attenuated in *Zdhhc21*^*dep/dep*^ mice. Quantitative analysis showed that septic injury resulted in significantly increased renal injury score, which was greatly inhibited by DHHC21 loss-of-function (Fig. 3b). The levels of circulating creatinine and blood urea nitrogen (BUN) were also measured as indicators of renal dysfunction. Consistent with the histology results, septic injury significantly up-regulated plasma levels of creatinine and BUN in WT mice; however, these changes were ameliorated in *Zdhhc21*^*dep/dep*^ mice (Fig. 3c,d).

We also compared the levels of circulating cytokines in WT and *Zdhhc21*^*dep/dep*^ mice after sham and septic injury. Cytokine array analysis revealed that CLP led to an increased production of cytokines such as interleukin-1 (IL-1), IL-6, and TNF-α in both WT and *Zdhhc21*^*dep/dep*^ mice; yet, there was no remarkable differences in cytokine profiles between the two mouse strains after CLP (Supplementary Fig. S2). These data indicate that the protective effects of DHHC21 functional deficiency on kidney function during septic injury are not due to inhibiting the production of pro-inflammatory cytokines.

### DHHC21 functional deficiency prevents the reduction of kidney perfusion and oxygen saturation during septic injury

**Figure 4:**
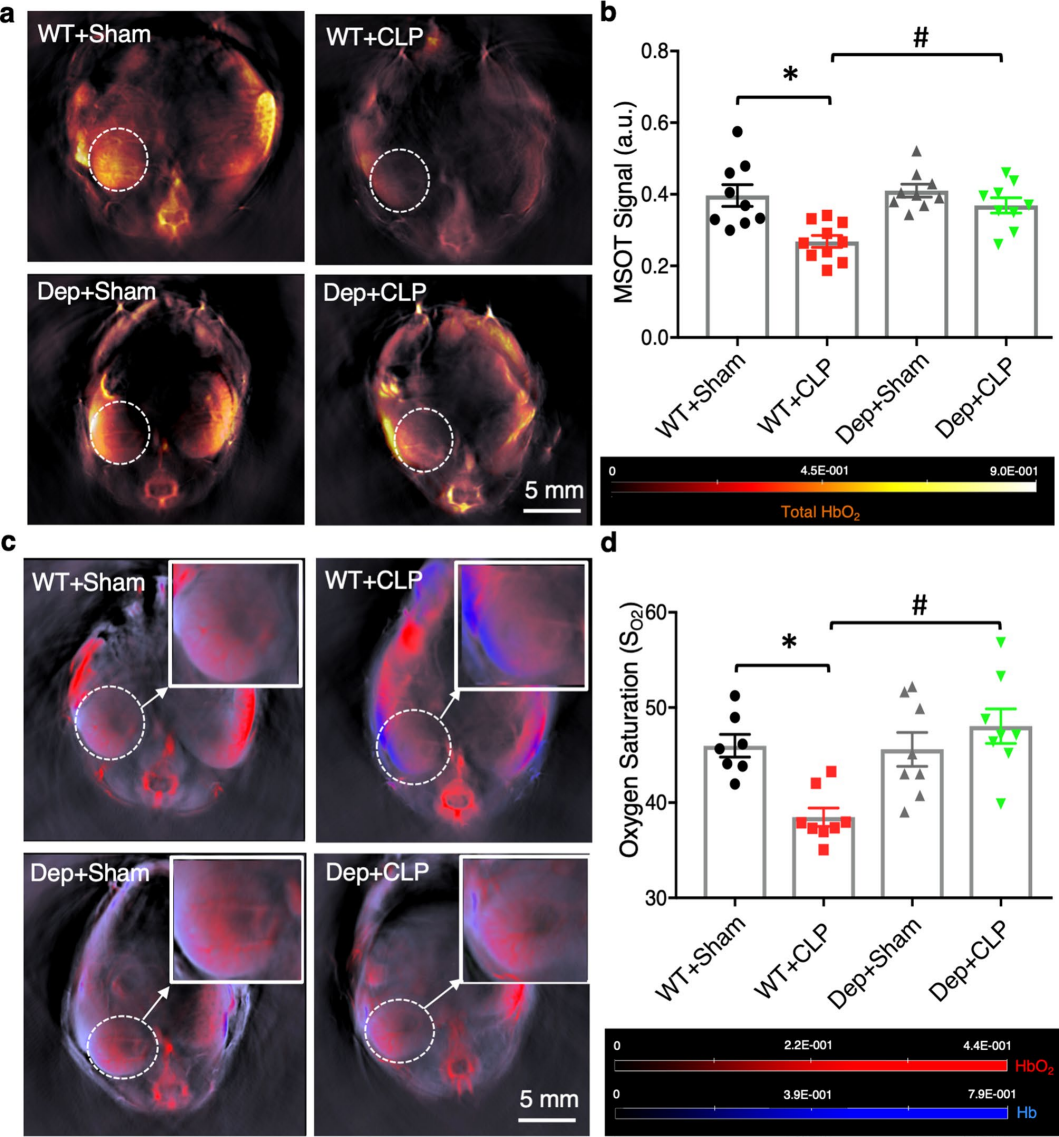
DHHC21 functional deficiency prevents the reduction of renal perfusion and renal oxygen saturation after septic injury. (**a**) Representative MSOT images showing the intensity of total hemoglobin in kidneys. MSOT images are pseudo-colored with hot-color scale. (**b**) Higher signal intensity of total hemoglobin is observed in kidneys of *Zdhhc21*^*dep/dep*^ mice than WT ones after septic injury. n = 9. (**c**) Reconstructed MSOT images indicate the distribution of oxygenated hemoglobin (red) and deoxygenated hemoglobin (blue) in kidney cross sections 24 h after CLP induction. (**d**) *Zdhhc21*^*dep/dep*^ mice display higher level of renal S_O2_ compared to WT mice after septic injury. ROIs are drawn around right kidneys. n = 8. Results represent mean ± SEM. *p ≤ 0.05 versus WT + Sham, ^#^p ≤ 0.05 versus WT + CLP.

Given the fact that renal tissue hypoperfusion is a major cause of renal dysfunction during septic injury, we compared kidney perfusion between WT and *Zdhhc21*^*dep/dep*^ mice after septic injury using MSOT. Detecting intrinsic NIR light absorbing hemoglobin via MSOT has been reported to be a reliable technique to measure organ perfusion in many studies^31,32^. As shown in Fig. 4a,b, the intensity of total hemoglobin was significantly decreased in kidneys of WT mice after CLP compared to sham controls. This reduction was greatly attenuated by DHHC21 functional deficiency, suggesting better-preserved renal perfusion in *Zdhhc21*^*dep/dep*^ mice after septic injury. Next, we differentiated oxygenated and deoxygenated hemoglobin based on their distinctive spectra and calculated the oxygen saturation of the renal tissue. After 24 h of CLP, WT mice displayed remarkably less oxygenated hemoglobin but greater deoxygenated hemoglobin in kidneys than sham-operated mice, whereas these responses were ameliorated in *Zdhhc21*^*dep/dep*^ mice (Fig. 4c). Consistently, quantitative data in Fig. 4d indicated that *Zdhhc21*^*dep/dep*^ mice were more resistant to septic injury-induced decrease in renal oxygen saturation.

### ***Zdhhc21***^***dep/dep***^ mice exhibit better-preserved RBF after septic injury

**Figure 5:**
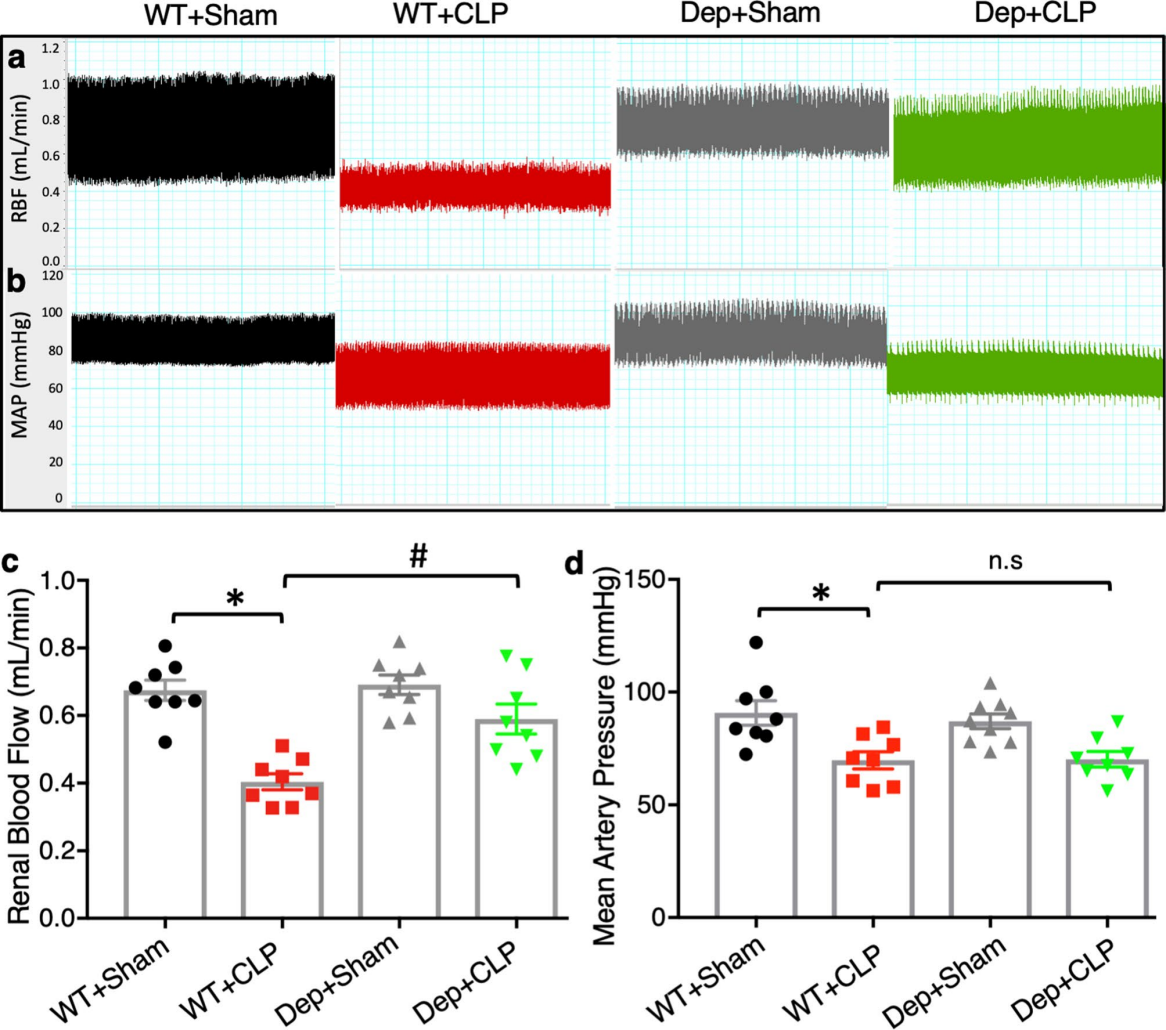
DHHC21 loss-of-function attenuates septic injury-induced reduction in renal blood flow. (**a**,**b**) Representative recordings of RBF (**a**) and MAP (**b**) in different groups 24 h after septic injury. Images are representative of 8 mice. (**c**) The reduced RBF in WT mice upon septic injury is rescued in *Zdhhc21*^*dep/dep*^ mice. n = 8 (**d**) No significant difference in MAP is observed between WT and *Zdhhc21*^*dep/dep*^ mice upon septic injury. n = 8. Results represent mean ± SEM. *p ≤ 0.05 versus WT + Sham, ^#^p ≤ 0.05 versus WT + CLP.

Next, we examined the functional impact of DHHC21 on RBF during septic injury. The levels of RBF of WT and *Zdhhc21*^*dep/dep*^ mice were compared using transonic flowmeter 24 h after CLP. Our results indicated that the RBF in WT mice was greatly reduced after septic injury; this reduction was significantly blunted by DHHC21 functional deficiency (Fig. 5a,c). We also monitored the changes in mean arterial pressure (MAP) during septic injury. As shown in Fig. 5b,d, septic injury caused a drastic decrease in MAP in WT mice; yet, no significant difference in MAP was found between WT and *Zdhhc21*^*dep/dep*^ mice after septic injury. These data indicate that the improved RBF in *Zdhhc21*^*dep/dep*^ mice is not secondary to systemic blood pressure changes.

### DHHC21 functional deficiency decreases α1AR palmitoylation and inhibits α1AR-mediated signaling pathway activation and vasoconstriction

**Figure 6:**
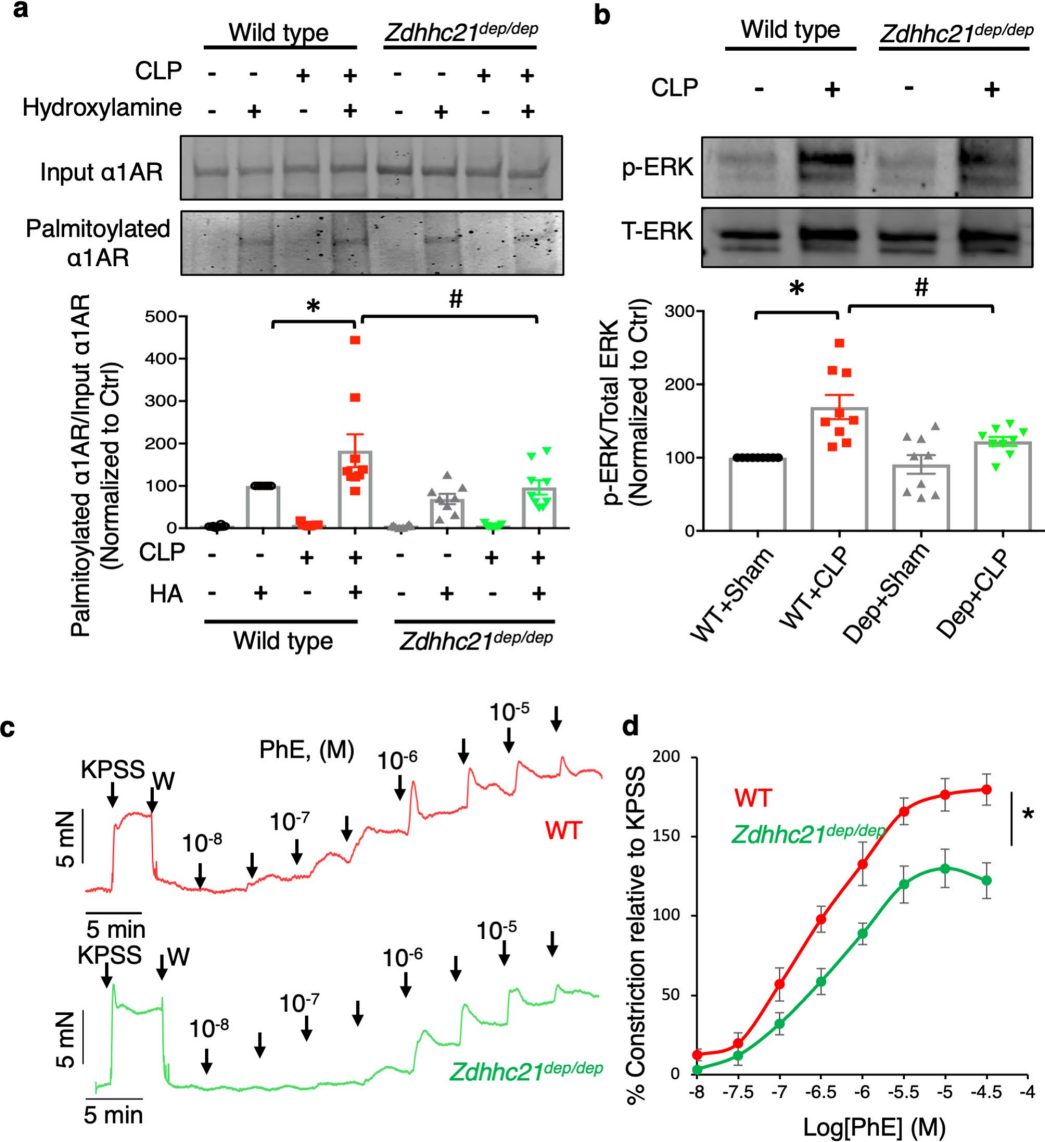
DHHC21 catalyzes α1AR palmitoylation and contributes to α1AR-mediated signaling pathway activation and vasoconstriction. (**a**) The level of palmitoylated α1AR in renal arteries. Palmitoylated proteins were isolated by RAC in the presence of hydroxylamine and then analyzed via immunoblotting for α1AR. Blots are representative of 8 independent experiments; renal arteries from 12 mice were pooled for each independent immunoblot analysis. Band intensity is quantified and normalized to control. Full-length blots are presented in Supplementary Fig. S5a. (**b**) Septic injury-induced ERK activation is inhibited in *Zdhhc21*^*dep/dep*^ mice. The band intensity of phosphorylated ERK is normalized to that of total ERK. n = 9. *p ≤ 0.05 versus WT + Sham, ^#^p ≤ 0.05 versus WT + CLP. Full-length blots are presented in Supplementary Fig. S5b. (**c**) Representative myograph recordings of WT and *Zdhhc21*^*dep/dep*^ renal arteries. KPSS represents 60 mM potassium physiological saline solution; W represents wash. (**d**) Myograph results show that renal arteries of *Zdhhc21*^*dep/dep*^ mice exhibit reduced tension compared to those of WT mice upon phenylephrine challenge. Results represent mean ± SEM. n = 9 mice. One artery per animal was used. *p ≤ 0.05 versus WT.

To investigate the mechanism by which DHHC21 regulates RBF and renal tissue perfusion, we determined the roles of DHHC21 in α1AR palmitoylation and function. Both DHHC21 and α1AR are localized in the plasma membrane, which allows DHHC21 to dynamically interact with α1AR. Moreover, α1AR has previously been reported to co-immunoprecipitate with DHHC21, indicating their direct binding interaction^15^. We compared the levels of α1AR palmitoylation in renal arteries of WT and *Zdhhc21*^*dep/dep*^ mice after CLP, using resin-assisted capture (RAC, Supplementary Fig. S3). As illustrated in Fig. 6a, septic injury resulted in a dramatic increase in α1AR palmitoylation in renal arteries of WT mice, which was significantly reduced in *Zdhhc21*^*dep/dep*^ mice. Furthermore, we assessed the phosphorylation of extracellular signal-regulated kinase (ERK), a downstream signaling event of α1AR activation, in renal arteries of WT and *Zdhhc21*^*dep/dep*^ mice after septic injury. The level of phosphorylated ERK was significantly increased by CLP in renal arteries of WT mice; this response was diminished by DHHC21 loss-of-function (Fig. 6b).

In an effort to further evaluate the functional impacts of DHHC21 on α1AR-mediated vasoconstriction, we isolated renal arteries from WT and *Zdhhc21*^*dep/dep*^ mice and challenged the vessels with phenylephrine, an α1AR agonist. Our wire myograph data showed no significant difference in the high potassium (60 mM)-induced vasoconstrictions between WT renal arteries and *Zdhhc21*^*dep/dep*^ renal arteries (Supplementary Fig. S4a); yet renal arteries isolated from *Zdhhc21*^*dep/dep*^ mice displayed a significant reduction in phenylephrine-induced vasoconstriction compared to those of WT mice (Fig. 6c,d). These results suggest the critical role of DHHC21-mediated α1AR palmitoylation in α1AR signaling activation and α1AR-mediated vasoconstriction.

**Figure 7:**
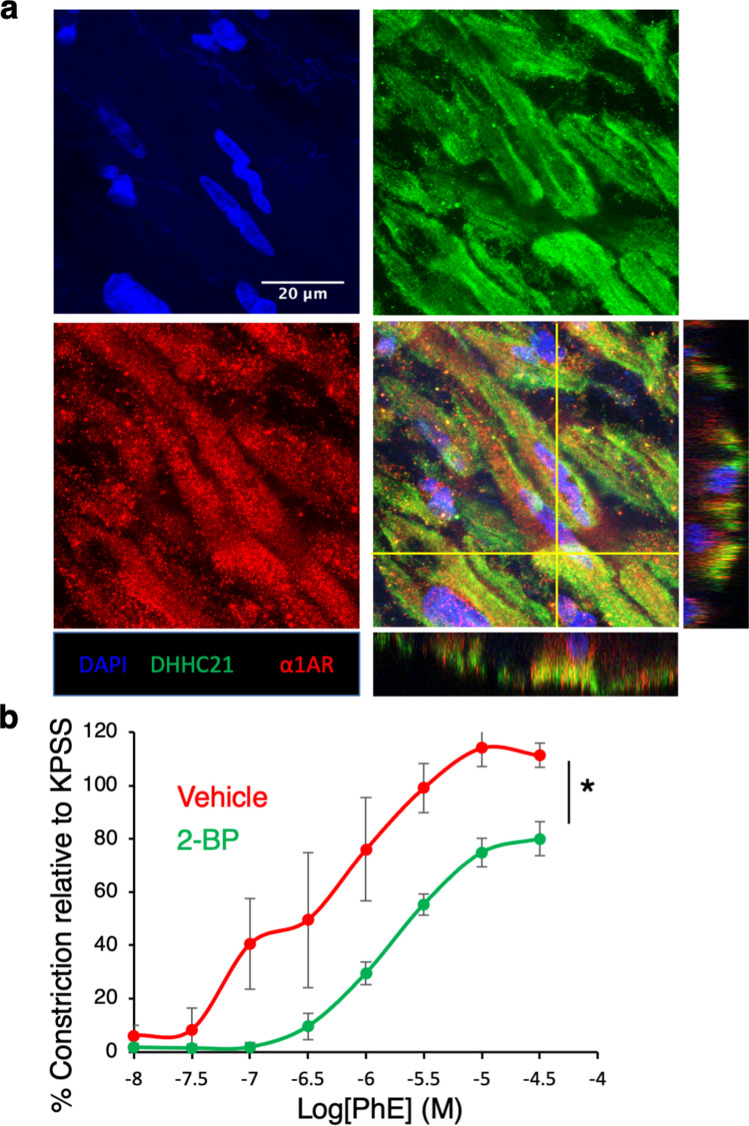
DHHC21 co-localizes with α1AR and palmitoylation regulates the constriction of small arteries isolated from human kidneys. (**a**) Co-localization of DHHC21 and α1AR in the cross-section of human renal arteries. Representative images of renal arteries isolated from 3 donors. (**b**) Inhibition of PATs with 2-BP results in impaired α1AR-mediated vasoconstriction in small arteries isolated from human kidneys. Vehicle control or 2-BP (100 μM) was given 1 h prior to phenylephrine challenge. Small arteries are from 3 different donors. Results represent mean ± SEM. *p ≤ 0.05.

Consistent findings were observed in small arteries isolated from viable human kidneys. Our immunofluorescence images showed evidence for co-localization of DHHC21 and α1AR in human renal arteries (Fig. 7a). PAT inhibitor 2-bromopalmitate (2-BP) was applied to block the function of DHHC21 in small arteries isolated from human kidneys. 2-BP treatment showed no effects on high potassium-induced vasoconstriction (Supplementary Fig. S4b); however, 2-BP-treated small arteries displayed blunted constriction responses to phenylephrine compared to vehicle-treated ones (Fig. 7b). Collectivity, the above findings demonstrated that DHHC21 contributes to impaired renal perfusion and function during septic injury via promoting α1AR palmitoylation and α1AR-mediated renal vasoconstriction.

## Discussion

In the present work, we report DHHC21 as a novel regulator of renal perfusion and function during septic injury. Our new findings indicate: (1) *Zdhhc21*^*dep/dep*^ mice exhibit better renal function and less renal damage after septic injury compared to WT mice; (2) DHHC21 functional deficiency preserves renal tissue perfusion, oxygen saturation, and RBF in septic injury; (3) DHHC21-catalyzed α1AR palmitoylation is required for the activation of α1AR signaling pathway and α1AR-induced constriction of renal arteries. This study provides a new mechanistic insight into the regulation of renal perfusion and function during septic injury. We suggest that DHHC21 functional deficiency confers protective effects on kidney function in septic injury and that inhibition of DHHC21 may serve as a therapeutic strategy to combat renal dysfunction during septic injury.

Renal tissue hypoperfusion/hypoxia is one of the leading causes of renal dysfunction during septic injury^3–5^. We detected renal hypoperfusion in mice after CLP, evidenced by weakened MSOT signal intensity of total hemoglobin, decreased oxygen saturation in renal tissue, and reduced RBF after septic injury. Consistent with our findings, the reduction in RBF has been observed in sepsis patients with kidney injury as well as in large-animal models of septic injury^7,10,11^. It is worth noting that several studies report that renal dysfunction can develop in sepsis animals with unchanged or even increased RBF^33,34^. These discrepant findings may be attributed to different animal models and methods used to measure blood flow. For instance, while CLP-induced polymicrobial septic injury has been commonly used, some studies use intravenous injection of *Escherichia coli*^35^.

Protein palmitoylation has recently been reported as a novel regulator of protein function, involved in the pathogenesis and progression of many diseases. It was first identified as a new type of post-translational modification by Schmidt et al.^36,37^. The discovery of the DHHC family had since promoted the rapid expansion of this field^38,39^. The roles of palmitoylation and PATs have been reported in numerous physiological and pathological conditions, including lipid metabolism, cancer, cardiovascular diseases, and neurological disorders^23,40,41^. Although the involvement of palmitoylation in polycystic kidney disease and kidney cancer has been previously reported^23,42^, there have been very limited studies examining the functional role of PATs in kidney diseases, specifically in renal dysfunction during septic injury. To the best of our knowledge, we are the first to investigate the role of DHHC21 in renal dysfunction in septic injury. Our findings indicate that the inhibition of DHHC21 greatly rescues kidney function and preserves kidney structure in septic injury.

Our study utilizing *Zdhhc21*^*dep/dep*^ mice demonstrates that the beneficial effects of DHHC21 functional deficiency on kidney function is attributed to its ability to improve renal tissue perfusion during septic injury. Under basal conditions, *Zdhhc21*^*dep/dep*^ mice show no signs of salt/water imbalance or renal structural/functional damage^15^. Yet, DHHC21 loss-of-function suppresses the septic injury-induced reduction of RBF, renal perfusion, and renal oxygen saturation. Increased renal vascular resistance caused by excessive renal vasoconstriction is considered as a major reason for impaired renal tissue perfusion in septic injury^6,9,33^. Our results indicate the involvement of α1AR in mediating septic injury-induced renal tissue hypoperfusion, as evidenced by the increase in α1AR palmitoylation and the activation of α1AR signaling pathway in septic injury. However, DHHC21 functional deficiency inhibits α1AR palmitoylation and results in a blunted ability of α1AR to activate its downstream effector and to mediate phenylephrine-induced vasoconstriction in renal arteries.

The molecular mechanism underlying the regulation of α1AR function by DHHC21 still remains to be fully elucidated. The defect in α1AR function caused by DHHC21 loss-of-function may be attributed to the conformational change of α1AR. Many GPCRs rely on palmitoylation for their appropriate intracellular conformation. The attached palmitate in the C-terminal tails of GPCRs inserts into the plasma membrane to create the fourth intracellular loop, which is essential for the interaction of GPCRs with their partner proteins and the propagation of GPCR signals^17,43^. Previous study has indicated that the palmitoylation of α1bAR also occurs in its C-terminal region^44^. Therefore, the lack of DHHC21-mediated α1AR palmitoylation may alter the intracellular conformation of α1AR, thus blocking the propagation of α1AR signals. This can be supported by our results showing that the activation of ERK, a downstream signaling event of α1AR activation, is inhibited in *Zdhhc21*^*dep/dep*^ mice subjected to septic injury. However, the DHHC21-regulated topology of α1AR carboxyl terminal needs to be further confirmed using X-ray crystallography.

Another novel aspect of our study is the utilization of MSOT to evaluate kidney perfusion and function during septic injury. MSOT has been reported as a label-free method to measure the perfusion of different organs and a reliable imaging technique to determine kidney function^28,32,45^. Compared to Doppler flowmeter which does not allow visualization of microvasculature^45^, MSOT generates images with high spatial resolution at 150 μm while providing quantitative assessment of the perfusion status in the kidney microvasculature. The water-soluble IRDye800CW is a safe tracer to use for measuring renal clearance, and it does not induce toxicity at the dose as high as 20 mg/kg^46^. The two-phase movement of IRDye800CW we observed is consistent with previously published studies^29^. Our MSOT recordings indicate a greater Tmax-delay in sepsis kidneys, suggesting impaired renal clearance. A nephropathy study demonstrates that the impaired renal function detected by MSOT is significantly correlated with glomerular histological damage^30^. Our data, consistent with previous publications, suggest that MSOT is a minimally-invasive and reliable technique to monitor renal perfusion and function.

Currently, there are no DHHC21-specific inhibitors available. The most commonly used PAT inhibitor is 2-BP which has a broad effect on multiple DHHCs and also interfere with fatty acid metabolism^47^. Thus, developing small molecule inhibitors that specifically target DHHC21 would be a promising direction. It is also worth pointing out that α1AR is not the only substrate of DHHC21. Several other DHHC21 substrates have recently been identified, including platelet endothelial cell adhesion molecule (PECAM-1), estrogen receptor, caveolin-1, endothelial nitric oxide synthase (eNOS), Fyn, superoxide dismutase (SOD-1), and PLCβ1^22,41,48,49^. While beyond the scope of the present study, we cannot rule out the possibility that these substrates may also affect renal function during septic injury.

In conclusion, the present study demonstrates for the first time that DHHC21 plays a critical role in regulating renal perfusion during septic injury via mechanism involving α1AR palmitoylation and α1AR-mediated vasoconstriction. Furthermore, inhibition of DHHC21 exerts protective effects on renal function during septic injury.

## Materials and methods

### Reagents

All reagents are listed in Supplementary Table S1.

### Animals

*Zdhhc21*^*dep/dep*^ mice and their wild-type control mice (B6C3Fe) were purchased from Jackson Laboratory. The genotype of *Zdhhc21*^*dep/dep*^ mice was confirmed by sequencing (Genewiz, Inc., NJ, USA). Primers used for sequencing were: AGCTGACTGAAGGGCACC (forward) and AAAACCTGTAACGCATTTCCA (reverse)^22^. Animals were maintained under a 12/12-h light/dark cycle with free access to food and water. Mice (16–20 weeks) of both genders were used for this study. All animal experiments are approved by the University of South Florida Institutional Animal Care and Use Committee and conform to the NIH Guide for the Care and Use of Laboratory Animals. The study was carried out in compliance with the ARRIVE guidelines.

### Cecal ligation and puncture

Mice were anesthetized with isoflurane (3% induction and 1% maintenance). A midline incision was made in the shaved abdominal region, and the cecum was exteriorized, tightly ligated at 5 mm below the ileocecal valve, and perforated twice with a 20-gauge needle distal to the point of ligation. One mm of feces was extruded from each puncture hole. The cecum was then repositioned, and the abdomen was closed in two layers. 37 °C Lactated Ringer solution was applied topically to prevent the cecum from drying. Mice were then given 37 °C 0.9% saline subcutaneously for fluid resuscitation. A preoperative 0.5–1.5 mg/kg dose of Buprenorphine Sustained-Release was given for analgesia. Sham mice were subjected to the same surgical procedure but without cecal ligation and puncture^50,51^.

### Multispectral optoacoustic tomography

#### Assessment of renal excretion of IRDye800CW

Mice were anesthetized via isoflurane and depilated around the torso region. Mice were imaged using iThera Medical MSOT Imaging System (Munich, Germany) at multiple wavelengths: 700, 730, 760, 775, 785, 800, 850 nm at a rate of 10 frames/s^29^. Mice were placed in a supine position in a holder and moved along the horizontal linear stage to position the kidneys on top of the fix-positioned concave transducer. After baseline recording, 60 nmol of IRDye800CW dissolved in 100 μl of 0.9% saline was injected intravenously over a period of 10 s. Images were reconstructed using the back-projection formula and processed with multispectral analysis. Regions of interest (ROIs) were drawn around the renal cortex and medulla/pelvis region, and the temporal changes of MSOT signal in ROIs were depicted.

#### Measurement of renal perfusion

Mice were prepared for MSOT imaging utilizing the same procedures mentioned above. Mouse respiration was closely monitored throughout the entire procedure. Images were reconstructed and the spectral unmixing was performed. ROIs were drawn around the entire right kidney region. Mean pixel intensities of oxygenated hemoglobin (HbO_2_) and deoxygenated hemoglobin (Hb) were acquired. Total hemoglobin (HbT = HbO_2_ + Hb) and oxygen saturation (S_O2_ = HbO_2_ /HbT) were then calculated^52^.

### Kidney histopathology

Kidneys were fixed, cut along the sagittal plane, and processed for paraffin-embedding. Sections (5 μm) were de-paraffinized, rehydrated, and oxidized with periodic acid for 8 min at room temperature (RT), followed by incubation with Schiff’s solution for 25 min. Sections were then counterstained with hematoxylin. Images were captured using Keyence BZ-X710 (Itasca, IL, USA). Renal structural damage was evaluated based on the following features: glomerular abnormality, loss of brush border of proximal tubule, vacuolation, dilation of tubule epithelium, tubular cell detachment/necrosis, and neutrophil infiltration^53^. Each feature was graded on a scale of 0–5 based on the severity.

### Measurement of creatinine and blood urea nitrogen

Mouse blood was collected via cardiac puncture 24 h after septic injury. Plasma was generated by centrifuging the blood at 2500 g for 15 min at RT. *Measurement of creatinine*: plasma was deproteinized using 10-kDa Spin Column; the levels of creatinine in the filtrate were then measured using a Creatinine Assay Kit. The level of blood urea nitrogen (BUN) was determined using a Urea Nitrogen Colorimetric Detection Kit following manufacturer’s instructions.

### Measurement of RBF and MAP

Mice were anesthetized using isoflurane. The blood pressure transducer was cannulated into the carotid artery. A midline incision was made in the abdominal area followed by a left transverse incision to expose the renal artery. RBF was measured using an ultrasound transit-time flowmeter (TS-420; Transonic Systems Inc., Ithaca, NY, USA). After placing the flow probe around the exposed renal artery, mice were allowed to be stabilized for at least 30 min. RBF and MAP were recorded simultaneously using PowerLab (AD Instruments, Colorado Springs, CO, USA). Data was analyzed using LabChart Pro version 7 software^54,55^.

### Resin-assisted capture

Renal arteries were collected and lysed in lysis buffer (100 mM HEPES, 25 mM NaCl, 1 mM EDTA, 10 μM palmostatin B, protease inhibitors, pH 7.4)^22,56^. Tissue lysates were incubated with blocking buffer (100 mM HEPES, 1 mM EDTA, 2.5% SDS, 6 μl/ml MMTS, pH 7.4) at 50 °C for 30 min with constant vortexing. After precipitating with cold acetone, proteins were pelleted by centrifugation at 5000*g* for 30 min and washed five times with 70% cold acetone. Protein pellets were resuspended in binding buffer (100 mM HEPES, 1.0% SDS, 1 mM EDTA, pH 7.4). Protein concentration was determined and normalized among groups. Each protein sample was divided into two equal parts which each received the same amount of thiopropyl sepharose 6B beads. Hydroxylamine (0.2 M, pH 7.4) and NaCl (0.2 M) were added into each part, respectively. After 4 h of incubation at RT with constant rotation, palmitoylated proteins were eluted with 50 mM DTT in 1 × Sample Buffer and collected for SDS-PAGE.

### Western blotting

#### Measurement of palmitoylated α1AR

Samples collected from RAC were loaded on a 4–20% Tris–Glycine gel and transferred onto a nitrocellulose membrane after electrophoresis. After blocking for 1 h at RT, α1AR was probed with rabbit anti-α1AR primary antibody (1:500) overnight at 4 °C. After washing three times with TBST, the membrane was incubated with IRDye800CW donkey anti-rabbit secondary antibody (1:20,000) for 45 min at RT.

#### Measurement of ERK phosphorylation

Renal arteries were lysed in 1 × RIPA containing protease and phosphatase inhibitors. Membrane was probed with rabbit anti-ERK (1:1000) and mouse anti-phosphorylated ERK (1:1000) antibodies. IRDye800CW donkey anti-mouse and IRDye680RD donkey anti-rabbit antibodies were used for secondary incubation (1:20,000). The membranes were imaged and analyzed using Li-COR Odyssey CLx.

### Assessment of vasoreactivity via wire myograph

#### Mouse renal arteries

Renal arteries (250–350 μm in diameter) were isolated from WT and *Zdhhc21*^*dep/dep*^ mice and submerged into ice-cold oxygenized (95% O_2_/5% CO_2_) physiological saline solution (PSS, 130 mM NaCl, 4.7 mM KCl, 1.18 mM KH_2_PO_4_, 1.17 mM MgSO_4_·7H_2_O, 14.9 mM NaHCO_3_, 5.5 mM glucose, 0.026 mM EDTA, and 1.6 mM CaCl_2_, pH 7.4). Vessel segments (~ 2 mm long) were mounted onto the wire myograph chamber (Living Systems Instrumentation, VT, USA) between two tungsten wires (30 μm in diameter). The isometric tension was recorded using Living Systems signal conditioner (MYO-SC-1) coupled with LabScribe v4 iWorks software. After equilibrating for 30 min at 37 °C in PSS, the normalization procedure was performed to determine optimal internal circumference *L*_1_ = 0.9 × *L*_100_ (*L*_100_ = internal circumference that corresponds to transmural pressure of 100 mmHg). Next, the vessel viability was tested with 60 mM KPSS (74.7 mM NaCl, 60 mM KCl, 1.18 mM KH_2_PO_4_, 1.17 mM MgSO_4_·7H_2_O, 14.9 mM NaHCO_3_, 5.5 mM glucose, 0.026 mM EDTA, 1.6 mM CaCl_2_, pH 7.4). Arteries that did not respond to KPSS were discarded. The arteries were treated with increasing concentrations of phenylephrine (10 nM–30 μM). Endothelial integrity was then assessed using acetylcholine (10 μM). The concentration–response curves were constructed.

#### Small arteries from human kidneys

Small arteries (~ 1000 μm in diameter) were dissected from intact viable human kidneys that were rejected for transplant surgery. Vessel segments (~ 2 mm long) were mounted onto the L-bars (250 μm in diameter) of wire myograph chamber. Similar equilibration and normalization procedures were performed on human arteries. Next, the arteries were incubated with vehicle control (0.02% DMSO in PSS) for 1 h. The vessels were then tested for viability with 60 mM KPSS and treated with increasing concentrations of phenylephrine (10 nM–30 μM). After washing off, the vessels were incubated with 2-BP (100 μM) for 1 h; the vessel viability was then tested again with 60 mM KPSS with 100 μM 2-BP. The small arteries with no or reduced responses to KPSS were discarded. The arteries were then challenged with the same concentrations of phenylephrine followed by acetylcholine (10 μM). The concentration–response curves of vehicle control- or 2-BP-treated arteries were constructed and compared.

### Immunofluorescence

Human renal arteries were fixed in 10% formalin for 48 h, paraffin-embedded and sectioned. Slides were then deparaffinized, rehydrated and permeabilized with PBS containing 0.05% Triton X-100^22^. After blocking, slides were labelled with rabbit anti-DHHC21 and goat anti-α1AR antibodies (1:100) overnight at 4 °C. After washing, secondary antibody incubation was done with donkey anti-rabbit Alexa Fluor 488 and donkey anti-goat Alexa Fluor 568 (1:500). After mounting with ProLong Diamond mounting medium with DAPI, slides were imaged with Leica SP8 spectral inverted laser scanning confocal microscope.

### Statistical analysis

Detailed information (e.g. the name of the test, post-hoc test, n value, alpha level, p value) for each statistical test is listed in Supplementary Table S2. All data meet the normal distribution assumption. Normality test was performed using Shapiro–Wilk Test with alpha level set at 0.05. Comparisons between two groups were analyzed using Student’s t test (two-tailed), and three groups or more were compared via One-way ANOVA with Tukey post-hoc analysis. The comparison for wire myograph curves was performed using Two-way ANOVA. p < 0.05 was used for statistical significance. All statistical analyses were performed using GraphPad Prism 7.0d (San Diego, CA, USA). The actual values for all quantification results are listed in Supplementary Table S3.

## Supplementary Information

Supplementary Information 1.

## Data Availability

The datasets generated and/or analyzed during the current study are available from the corresponding author on reasonable request.
