# Supplementary material for: DHHC21 deficiency attenuates renal dysfunction during septic injury
Source: Sci Rep. 2021 May 27;11:11146. doi: 10.1038/s41598-021-89983-x (PMC8159935; doi:10.1038/s41598-021-89983-x)
Supplement: Supplementary file 1 — Supplementary Information 1. [file 41598_2021_89983_MOESM1_ESM.pdf]

DHHC21 Deficiency Attenuates Renal Dysfunction During Septic Injury

Xiaoyuan Yang[1], Ethan Zheng[1], Yonggang Ma[1], Victor Chatterjee[1], Nuria Villalba[1], Jerome W. Breslin[1], Ruisheng Liu[1], Sarah Y. Yuan [1,2*]

Supplementary Figure 1

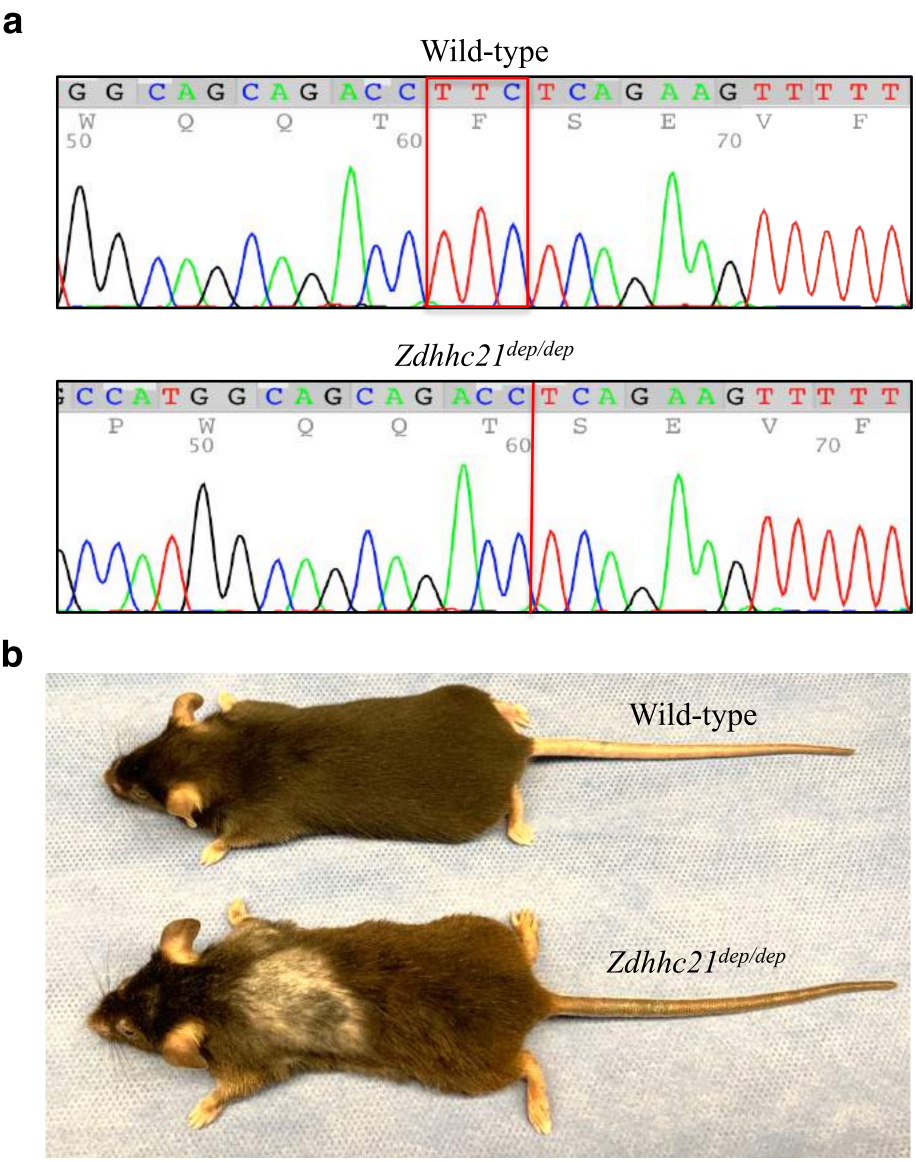

Supplementary Figure 2

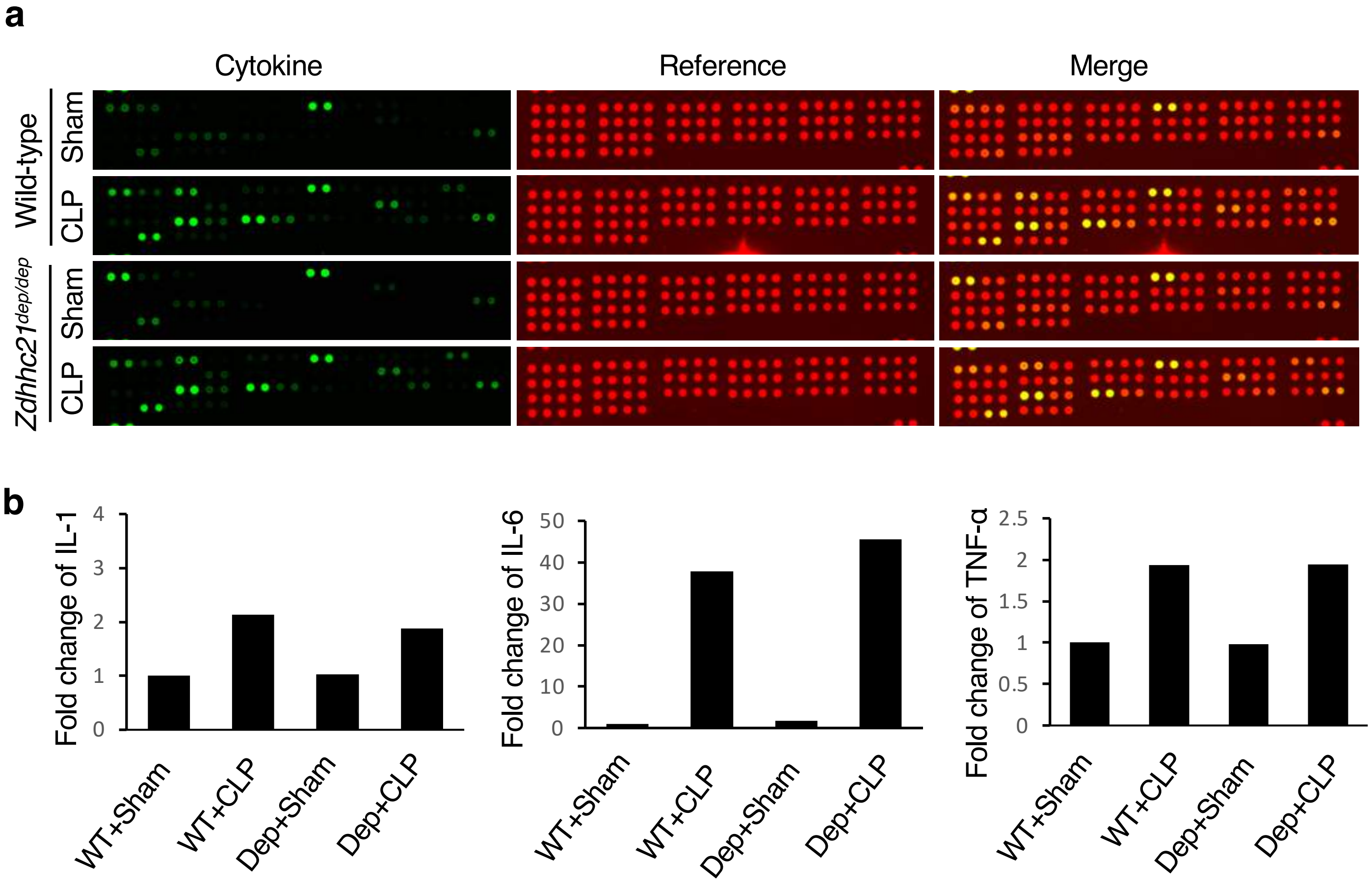

Supplementary Figure 3

a

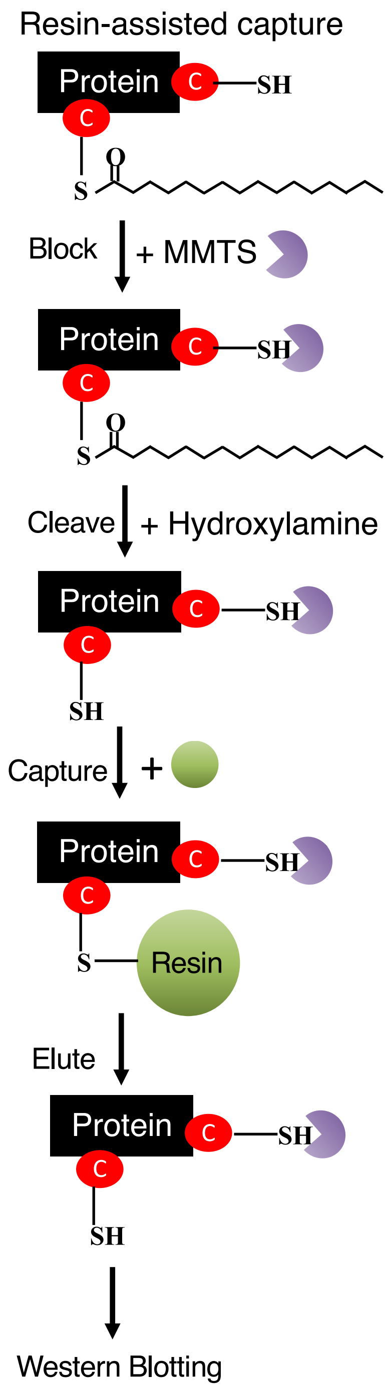

Supplementary Figure 4

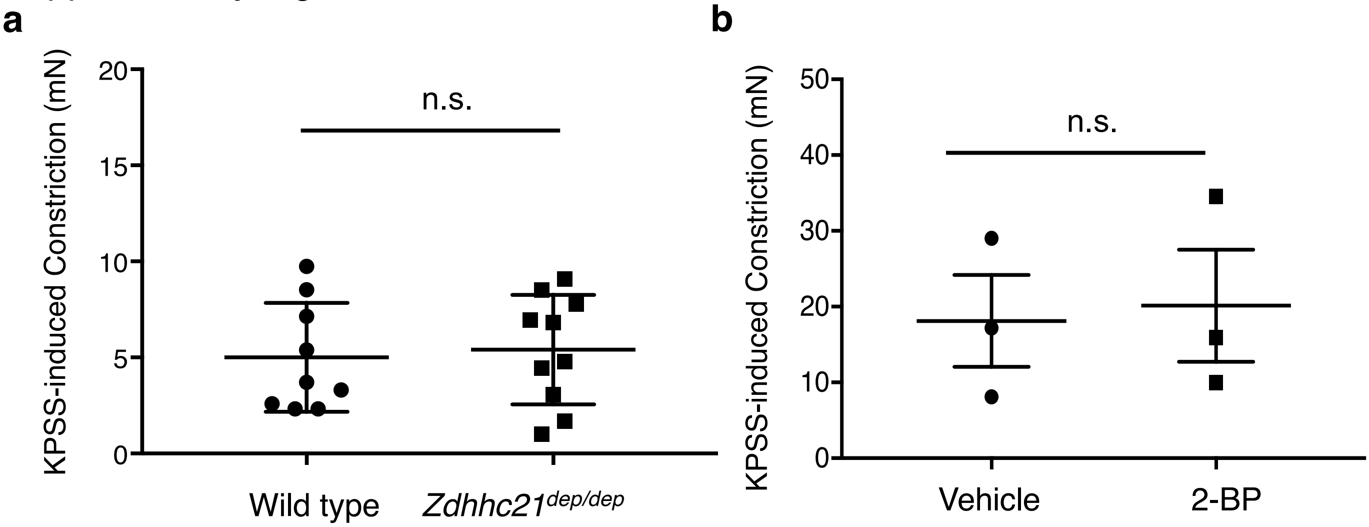

Supplementary Figure 5

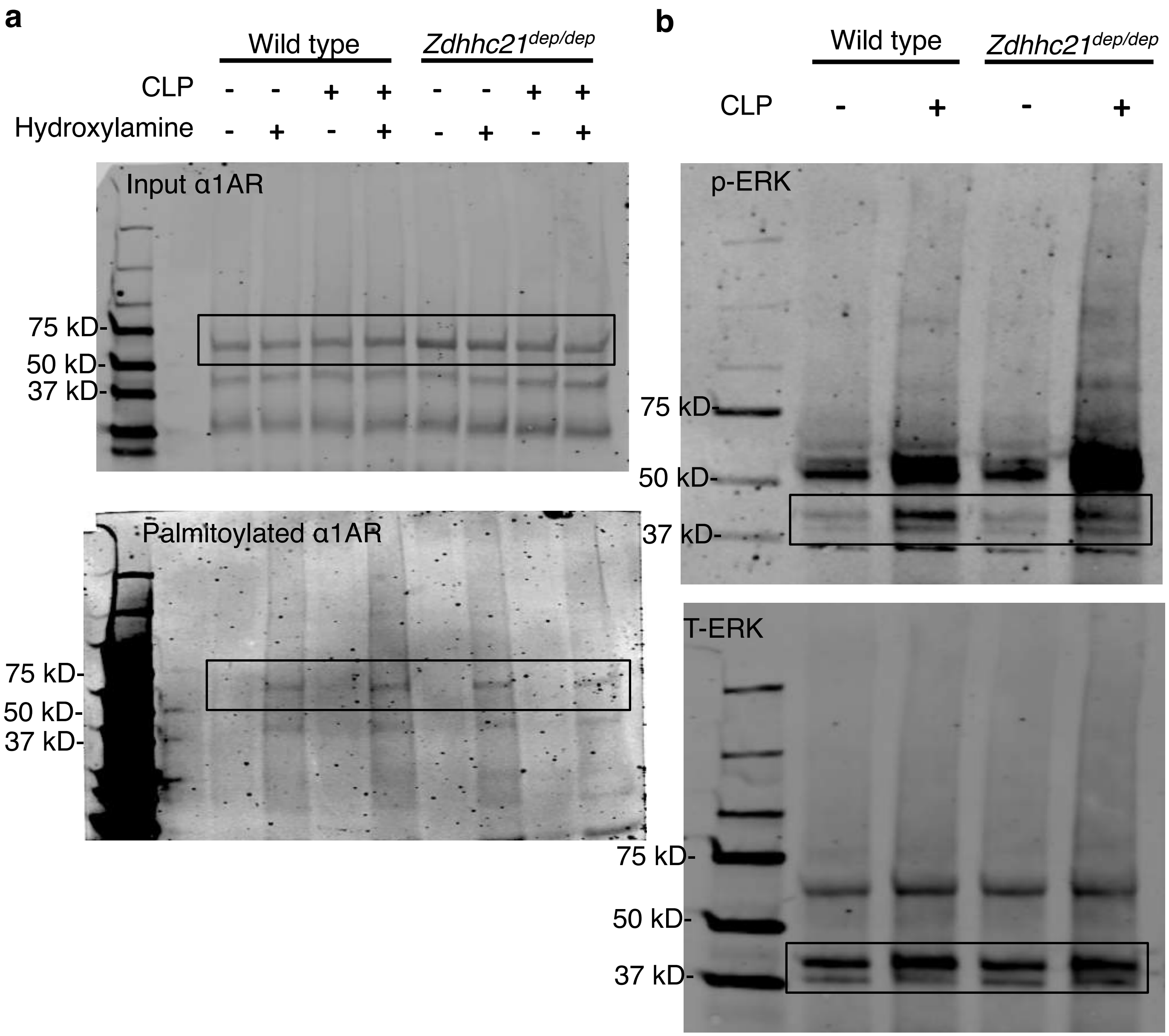

Supplementary Figure Legend

Supplementary Figure 1. The genotype and phenotype of *Zdhhc21* $^{dep/dep}$ mice. a. Sequencing of the *Zdhhc21* gene reveals a 3-bp deletion (nucleotides 1061-1063 within exon 7) in *Zdhhc21*$^{dep/dep}$ mice, resulting in the deletion of phenylalanine 233 in DHHC21. **b.** The phenotype of *Zdhhc21*$^{dep/dep}$ mice is characterized by hair loss and greasy pigmented skin.

Supplementary Figure 2. Cytokine profile of WT and *Zdhhc21*$^{dep/dep}$ mice in septic injury. a. Following sham or CLP procedure, mouse plasma was collected and analyzed using mouse cytokine array kit. For each group, plasma from 6 mice were pooled. **b.** Quantification of signal intensity shows comparable levels of IL-1, IL-6, and TNF-α in plasma of WT and *Zdhhc21*$^{dep/dep}$ mice 24 hours after septic injury.

Supplementary Figure 3. Schematic image of resin-assisted capture. The free thiol groups of protein are blocked by methylmethanethiosulfonate (MMTS). Next, palmitoylated proteins are cleaved by hydroxylamine to liberate the previously palmitoylated cysteinyl thiol groups. The newly freed thiol groups are then captured by thiol-reactive Sepharose resin. The captured proteins are eluted by DTT for Western Blotting.

Supplementary Figure 4. Vasoconstriction induced by high potassium. a. There is no significant difference in 60 mM KPSS-induced vasoconstriction between WT renal arteries and *Zdhhc21*$^{dep/dep}$ renal arteries. n=9. **b**. 2-BP (100 μM) does not cause a significant change in 60 mM KPSS-induced vasoconstriction in small arteries isolated from human kidneys. n.s. =no significance.

Supplementary Figure 5. Full-length Western Blots. a. Full-length blots for Fig 6a. **b**. Full-length blots for Fig 6b.

SUPPLEMENTARY TABLE S1

Reagent/Drug	Company	Catalogue Number
2-BP	Sigma	238422
4×Protein Loading Buffer	Li-Cor	928-40004
10kD Spin Column	Abcam	ab93349
Acetylcholine Chloride	Sigma	A6625
Acetone	Fisher	A181
BCA Assay	Fisher	PI23227
β-mercaptoethanol	MP Biomedicals LLC	194834
Citrate Antigen Retrieval Buffer	Abcam	ab93678
Creatinine Assay kit	Abcam	ab65340
Donkey Anti-rabbit AlexaFluor 488	Invitrogen	A21206
Donkey Anti-goat AlexaFluor 568	Invitrogen	A11057
Donkey Serum	Abcam	ab7475
DTT	Fisher	BP172
Goat anti-α1AR Primary Antibody	Abcam	ab166925
Hydroxylamine (HA)	Sigma	159417
IRDye 800 CW	Li-Cor	929-09406
IRDye 680RD Donkey Anti-Mouse	Li-Cor	926-68072
IRDye 680RD Donkey Anti-Rabbit	Li-Cor	926-68073
IRDye 800CW Donkey Anti-Mouse	Li-Cor	926-32212
IRDye 800CW Donkey Anti-Rabbit	Li-Cor	926-32213
Isoflurane	Piramal Healthcare	NDC 66794-017-25
iQ SYBR Green Supermix	Bio-rad	170-8880AP
iScript cDNA Synthesis kit	Bio-rad	170-8891
Lactated Ringer's Injection, USP	Hospira	0409-7953-03
Lanthanum Nitrate Hexahydrate	Electron Microscopy Sciences	17300
Methylmethanethiosulfonate (MMTS)	Sigma	208795
Mouse Anti-Phosphorylated ERK Primary Antibody	CST	9106s
Odyssey Protein Molecular Weight Marker	Li-Cor	928-40000
Palmostatin B (ATP1 inihibitor)	EMD Millipore	178501
Phenylephrine	Sigma-Aldrich	P6126-10G
PrimePCR *zdhhc21* primer	Bio-rad	qMmuCED0048001
PrimePCR *Gapdh* primer	Bio-rad	qMmuCED0027497
ProLong Diamond Antifade Mountant with DAPI	Life Technologies	P36962
PureLink RNA Mini Kit	Thermo Fisher	12183020
Periodic Acid Schiff staining kit	abcam	ab150680
Rabbit anti-α1AR Primary Antibody	abcam	ab3462
Rabbit anti-DHHC21 Primary Antibody	Fisher Scientific	PA5-25096
Rabbit anti-ERK Primary Antibody	CST	4695s
RIPA	EMD Millipore	20-188
Thiopropyl Sepharose 6B	GE Healthcare	17-0420-17
Urea Nitrogen Colorimetric Detection Kit	Invitrogen	EIABUN

SUPPLEMENTARY TABLE S2

Figure Panel	n	Statistical Test	Alpha leve	Post-hoc Test	P value
Fig. 2b	7	One-way ANOVA	0.05	Tukey’s	* p=0.0007 vs. WT+sham; # p=0.0028 vs. WT+CLP
Fig. 3b	5	One-way ANOVA	0.05	Tukey’s	* p<0.0001 vs. WT+Sham; # p<0.0001 vs.WT+CLP
Fig. 3c	12	One-way ANOVA	0.05	Tukey’s	* p=0.0002 vs. WT+sham; # p=0.0035 vs. WT+CLP
Fig. 3d	11	One-way ANOVA	0.05	Tukey’s	* p=0.0003 vs. WT+Sham; # p=0.0023 vs.WT+CLP
Fig. 4b	9	One-way ANOVA	0.05	Tukey’s	* p=0.0011 vs. WT+sham; # p=0.0126 vs. WT+CLP
Fig. 4d	8	One-way ANOVA	0.05	Tukey's	* p=0.0093 vs. WT+Sham; # p=0.0005 vs.WT+CLP
Fig. 5c	8	One-way ANOVA	0.05	Tukey’s	* p<0.0001 vs. WT+sham; # p=0.021 vs. WT+CLP
Fig. 5d	8	One-way ANOVA	0.05	Tukey’s	* p=0.0058 vs. WT+Sham; ns. p=0.9998 vs.WT+CLP
Fig. 6a	8	One-way ANOVA	0.05	Tukey’s	* p=0.0054 vs. WT+sham; # p=0.045 vs. WT+CLP
Fig. 6b	9	One-way ANOVA	0.05	Tukey’s	* p=0.0127 vs. WT+Sham; # p=0.0327 vs.WT+CLP
Fig. 6d	9	Two-way ANOVA	0.05	N/A	* p<0.0001
Fig. 7b	3	Two-way ANOVA	0.05	N/A	* p<0.0001
Fig. S4a	9	T-test (two-tailed)	0.05	N/A	ns. P=0.7597
Fig. S4b	3	T-test (two-tailed)	0.05	N/A	ns. P=0.8429

SUPPLEMENTARY TABLE S3

Figure Panel	mean±SEM			mean±SEM		mean±SEM		mean±SEM	
**Fig. 2b**	WT+Sham: 35.86±7.24			WT+CLP: 99.29±18.38		Dep+Sham: 34.14±3.36		Dep+CLP: 43.57±11.97	
**Fig. 3b**	WT+Sham: 3.68±0.64			WT+CLP: 15.44±1.51		Dep+Sham: 3.4±0.62		Dep+CLP: 43.57±11.97	
**Fig. 3c**	WT+Sham: 80.37±7.12			WT+CLP: 176.73±26.14		Dep+Sham: 59.93±4.55		Dep+CLP: 103.00±4.72	
**Fig. 3d**	WT+Sham: 14.08±0.89			WT+CLP: 75.11±17.42		Dep+Sham: 16.45±0.49		Dep+CLP: 24.86±4.02	
**Fig. 4b**	WT+Sham: 0.40±0.03			WT+CLP: 0.27±0.02		Dep+Sham: 0.41±0.02		Dep+CLP: 0.37±0.02	
**Fig. 4d**	WT+Sham: 45.99±1.42			WT+CLP: 38.48±0.97		Dep+Sham: 45.60±1.60		Dep+CLP: 48.06±1.81	
**Fig. 5c**	WT+Sham: 0.67±0.03			WT+CLP: 0.40±0.02		Dep+Sham: 0.69±0.03		Dep+CLP: 0.59±0.04	
**Fig. 5d**	WT+Sham: 90.75±5.47			WT+CLP: 69.78±3.79		Dep+Sham: 87.00±3.24		Dep+CLP: 70.23±3.63	
	WT+Sham			WT+CLP		Dep+Sham		Dep+CLP	
**Fig. 6a**	HA- 4.47±0.79		HA+ 100.00±0	HA- 8.32±1.40	HA+ 183.00±38.80	HA- 4.77±1.03	HA+ 69.52±12.14	HA- 5.40±1.11	HA+ 96.60±16.80
**Fig. 6b**	WT+Sham: 100.00±0			WT+CLP: 169.22±16.47		Dep+Sham: 90.91±12.71		Dep+CLP: 122.319±6.08	
		10^{-8}	$10^{-7.5}$	10^{-7}	$10^{-6.5}$	10^{-6}	$10^{-5.5}$	10^{-5}	$10^{-4.5}$
	WT	12.48±3.74	20.03±6.27	56.99±10.48	97.98±8.12	132.80±13.60	165.99±8.22	176.32±10.15	179.73±9.93
**Fig. 6d**	Dep	3.28±1.03	12.20±6.26	32.12±7.06	58.86±8.22	88.86±6.67	119.99±11.64	130.02±11.96	122.35±11.12
		10^{-8}	$10^{-7.5}$	10^{-7}	$10^{-6.5}$	10^{-6}	$10^{-5.5}$	10^{-5}	$10^{-4.5}$
	Ctrl	6.15±3.89	8.44±8.16	40.50±17.01	49.52±25.30	75.98±19.39	99.07±9.13	114.08±7.02	111.32±4.50
**Fig. 7b**	2-BP	1.77±1.53	1.48±1.42	1.83±1.78	9.64±4.95	29.51±4.35	55.35±3.89	74.84±5.30	80.00±6.42
**Fig. S4a**	WT: 5.01 ± 0.94					Dep: 5.41 ± 0.90			
**Fig. S4b**	Ctrl: 18.12±6.06					2-BP: 20.14±7.39			
